# Approaches to Ventral Hernia Repair: A Narrative Review

**DOI:** 10.7759/cureus.111008

**Published:** 2026-06-17

**Authors:** Naren Kumar A, Chebolu Mahalakshmi Eswar, Gokul D Yatheendranathan, Sankar Gopi C

**Affiliations:** 1 Department of General Surgery, Shri Sathya Sai Medical College and Research Institute, Sri Balaji Vidyapeeth (Deemed to be University), Chennai, IND

**Keywords:** hernia, laparoscopy, mesh repair, narrative review, ventral hernia

## Abstract

Ventral hernia repair is a significant and evolving challenge in general surgery, encompassing a wide spectrum of primary and incisional abdominal wall defects of varying complexity. The burden of ventral hernia continues to rise globally due to increasing rates of abdominal surgeries, aging populations, and the growing prevalence of risk factors such as obesity and metabolic disorders. Despite advances in surgical techniques, recurrence and postoperative complications remain important concerns, necessitating continuous refinement of management strategies. This narrative review synthesizes contemporary evidence on ventral hernia repair, focusing on mesh-based techniques, classification systems, patient optimization, and evolving surgical approaches.

Mesh reinforcement remains the cornerstone of modern hernia repair, with strong evidence supporting the superiority of synthetic mesh over biologic mesh for recurrence outcomes, even in contaminated settings. Advances in mesh design, particularly the adoption of lightweight materials, have improved patient-reported outcomes by reducing chronic pain without compromising durability. Patient-related factors such as obesity significantly influence perioperative outcomes, highlighting the importance of individualized treatment strategies.

The concept of loss of domain has emerged as a critical determinant of surgical complexity, with imaging-based volumetric assessment playing a key role in preoperative planning. Prehabilitation and multidisciplinary optimization have gained prominence as essential components of care. Evidence demonstrates that structured preoperative interventions, including exercise, nutritional support, and risk factor modification, reduce complications and improve recovery. Adjunct techniques such as botulinum toxin injection and progressive pneumoperitoneum further enhance outcomes in complex hernia repair by facilitating tension-free closure.

Minimally invasive approaches, including laparoscopic and robotic techniques, have transformed ventral hernia surgery. Laparoscopic repair is associated with reduced wound complications and shorter hospital stays compared to open repair while maintaining similar recurrence rates. Robotic surgery offers additional technical advantages and improved short-term outcomes, although concerns regarding operative time and cost remain. Advanced techniques such as enhanced-view totally extraperitoneal repair and transversus abdominis release have expanded the surgical armamentarium for complex cases.

Perioperative strategies continue to evolve, with emerging evidence questioning the routine use of surgical drains. Overall, ventral hernia repair has transitioned toward a patient-centered, evidence-based approach that integrates surgical innovation with perioperative optimization. Future research should focus on long-term outcomes, cost-effectiveness, and personalized treatment strategies to further improve patient care.

## Introduction and background

Ventral hernias represent one of the most frequently encountered disorders of the abdominal wall in contemporary surgical practice and continue to pose a major clinical and reconstructive challenge worldwide. These hernias encompass a heterogeneous group of defects involving the anterior abdominal fascia and musculature, including primary ventral hernias such as umbilical, epigastric, Spigelian, and lumbar hernias, as well as incisional hernias that develop following abdominal surgical procedures. The pathogenesis of ventral hernia formation is multifactorial and involves a complex interplay between impaired fascial integrity, altered collagen metabolism, increased intra-abdominal pressure, patient-related comorbidities, and technical factors associated with abdominal wall closure [1]. Although traditionally regarded as a purely mechanical defect, contemporary evidence increasingly recognizes ventral hernia disease as a systemic disorder influenced by biological, metabolic, and perioperative determinants that collectively affect tissue healing, recurrence, and postoperative outcomes [2].

Narrative reviews play a critical role in synthesizing evolving evidence across broad clinical domains, particularly in surgical specialties where heterogeneity of operative techniques, patient selection, and outcome reporting often limits the feasibility of conventional meta-analytic approaches [3]. Unlike systematic reviews that focus on narrowly defined research questions, narrative reviews provide a flexible yet academically rigorous framework for integrating diverse evidence, contextualizing findings, and identifying emerging trends in clinical practice. This approach is especially valuable in the field of abdominal wall reconstruction, where rapid technological innovation, evolving biomaterials, expanding minimally invasive platforms, and increasing procedural complexity necessitate continual reassessment of current evidence and treatment paradigms.

The global burden of ventral hernia has increased substantially over the past two decades. Rising rates of abdominal surgery, increasing life expectancy, escalating prevalence of obesity and metabolic syndrome, and greater survival following major intra-abdominal procedures have collectively contributed to the growing incidence of ventral and incisional hernias [4]. Incisional hernias are particularly important because they remain among the most common long-term complications following laparotomy. Current literature estimates that approximately 10-20% of patients undergoing open abdominal surgery eventually develop an incisional hernia, with substantially higher rates reported among high-risk populations such as obese individuals, smokers, diabetic patients, and those with wound infections or repeated abdominal surgeries [4,5]. Furthermore, the true incidence may be underestimated because many patients remain asymptomatic during the early stages of disease progression.

Beyond their anatomical manifestation, ventral hernias significantly impair patient quality of life and functional capacity. Patients frequently experience chronic pain, abdominal wall weakness, cosmetic deformity, impaired mobility, exercise intolerance, and psychological distress. In severe cases, hernias may lead to incarceration, bowel obstruction, strangulation, respiratory compromise, and enterocutaneous fistula formation, all of which contribute to substantial morbidity and occasional mortality [6]. The socioeconomic burden is equally considerable. Ventral hernia repair constitutes one of the most commonly performed procedures in general surgery, accounting for significant healthcare expenditure due to recurrent admissions, prolonged hospitalization, reoperations, mesh-related complications, and long-term postoperative surveillance [7]. As healthcare systems increasingly emphasize value-based care, optimization of ventral hernia management has become an important priority in modern surgical practice.

The biological basis of ventral hernia formation has also been better elucidated in recent years. Alterations in collagen metabolism, particularly abnormalities in the ratio of type I to type III collagen, have been implicated in impaired fascial strength and defective wound healing [8]. Matrix metalloproteinase dysregulation, impaired fibroblast function, chronic inflammation, and connective tissue remodeling further contribute to weakening of the abdominal wall. Patient-specific factors such as obesity, smoking, diabetes mellitus, chronic pulmonary disease, malnutrition, steroid therapy, and immunosuppression exacerbate these mechanisms by impairing tissue oxygenation and collagen synthesis [9]. Increased intra-abdominal pressure associated with obesity, ascites, chronic cough, constipation, or pregnancy further predisposes to fascial disruption and hernia progression. Consequently, contemporary ventral hernia management extends beyond operative repair alone and increasingly incorporates comprehensive risk stratification and perioperative optimization.

Substantial progress has been achieved in the prevention and management of ventral hernias through refinement of abdominal wall closure techniques and improved understanding of surgical biomechanics. International guidelines from the European Hernia Society and other expert groups emphasize evidence-based approaches to fascial closure aimed at reducing the incidence of postoperative incisional hernia formation [10]. Techniques such as small-bite closure, appropriate suture-to-wound length ratios, continuous slowly absorbable sutures, and tension-minimized fascial approximation have demonstrated significant reductions in incisional hernia rates compared to traditional closure methods [10]. The concept of prophylactic mesh augmentation in high-risk patients has also gained increasing attention, particularly in obese patients and those undergoing emergency laparotomy or abdominal aortic aneurysm repair [11].

The introduction of prosthetic mesh reinforcement has significantly improved ventral hernia repair outcomes, with lower recurrence rates and greater durability than primary suture repair [12]. Synthetic meshes remain the standard of care in most settings, while biologic and biosynthetic meshes may be considered in selected complex or contaminated cases [13]. Advances in mesh design, particularly lightweight materials, have further improved patient outcomes by reducing chronic pain and foreign-body sensation.

Advances in operative techniques have expanded the management options for ventral hernias. While open repair remains essential for large and complex defects, minimally invasive approaches are increasingly preferred because of reduced wound complications, postoperative pain, and hospital stay [14]. Robotic-assisted surgery has further enhanced abdominal wall reconstruction by enabling precise dissection and advanced intracorporeal suturing, thereby broadening the scope of minimally invasive hernia repair [15].

Concurrently, improved understanding of abdominal wall anatomy and biomechanics has led to the development of advanced reconstructive techniques for complex hernias. Retromuscular mesh placement, component separation methods, enhanced-view totally extraperitoneal (eTEP) repair, and TAR have enabled restoration of abdominal wall function while minimizing tension and recurrence [16]. The modern concept of abdominal wall reconstruction, therefore, extends beyond simple fascial closure and increasingly emphasizes restoration of functional anatomy, preservation of musculofascial integrity, and optimization of patient-centered outcomes.

Standardized classification systems have improved communication and surgical planning in ventral hernia management. The International Classification of Abdominal Wall Planes (ICAP) and consensus definitions for complex hernias provide structured frameworks for describing operative anatomy and disease severity [17,18]. In addition, loss of domain (LOD) has emerged as an important predictor of operative complexity, with computed tomography-based volumetric assessment playing a key role in preoperative planning [19].

Perioperative optimization has become an integral component of modern ventral hernia management. Structured preoperative interventions, including risk-factor modification, nutritional support, and exercise-based prehabilitation, have been shown to improve outcomes and reduce complications [20]. Adjuncts such as botulinum toxin A injection and progressive pneumoperitoneum further facilitate tension-free closure in complex hernias with significant loss of domain [21].

Despite these advances, ventral hernia repair continues to be associated with significant challenges. Recurrence, chronic pain, mesh infection, seroma formation, enterotomy, fistula development, and surgical site occurrences remain major concerns, particularly in complex reconstructions [22]. Considerable controversy persists regarding optimal mesh selection, ideal operative plane, role of biologic materials, indications for robotic surgery, use of drains, and cost-effectiveness of emerging technologies. Furthermore, long-term outcome data for several newer techniques remain limited. As a result, ventral hernia surgery continues to evolve rapidly, driven by ongoing innovation in biomaterials, imaging, minimally invasive technology, and perioperative care.

In this context, the present narrative review aims to provide a comprehensive and contemporary overview of ventral hernia repair, integrating current evidence regarding pathophysiology, classification systems, mesh science, minimally invasive approaches, reconstructive techniques, perioperative optimization, and emerging innovations in abdominal wall surgery. By synthesizing recent literature and highlighting ongoing controversies and future directions, this review seeks to provide clinicians with an updated understanding of the evolving landscape of ventral hernia management.

Methodology

This narrative review was conducted through a comprehensive literature search of PubMed, Scopus, Google Scholar, and Cochrane Library databases. Relevant articles published in English were identified using combinations of keywords including “ventral hernia,” “incisional hernia,” “hernia repair,” “mesh repair,” “laparoscopic ventral hernia repair,” “robotic ventral hernia repair,” “component separation,” and “abdominal wall reconstruction.” Priority was given to recent systematic reviews, meta-analyses, randomized controlled trials, society guidelines, and landmark studies addressing surgical techniques, perioperative optimization, and outcomes in ventral hernia repair. The selected literature was narratively synthesized to provide a contemporary overview of current approaches and emerging concepts in ventral hernia management.

## Review

Prehabilitation and multidisciplinary optimization

Prehabilitation has emerged as an increasingly important component in the contemporary management of ventral hernia patients, particularly among individuals with large defects, recurrent hernias, obesity, frailty, or multiple metabolic comorbidities. Traditionally, ventral hernia surgery focused primarily on technical operative repair; however, modern evidence demonstrates that optimization of physiological reserve and modification of risk factors before surgery significantly influence postoperative outcomes, recurrence rates, and overall patient recovery [17]. The concept of prehabilitation involves structured interventions implemented during the preoperative period to improve a patient’s functional, nutritional, metabolic, and psychological status prior to surgery. This paradigm reflects the broader shift toward patient-centered and multidisciplinary abdominal wall reconstruction.

Several patient-related factors adversely affect outcomes following ventral hernia repair. Obesity, diabetes mellitus, smoking, chronic pulmonary disease, malnutrition, sarcopenia, and poor cardiovascular reserve are independently associated with increased risks of surgical site infection, seroma formation, respiratory complications, delayed wound healing, mesh infection, and recurrence [18]. Consequently, optimization of these modifiable risk factors has become a central component of perioperative planning in specialized abdominal wall reconstruction programs.

A systematic review by Jensen et al. demonstrated that structured prehabilitation interventions, including supervised exercise programs, smoking cessation, respiratory training, nutritional supplementation, and metabolic optimization, significantly reduce postoperative complications while improving functional recovery and patient-reported outcomes [17]. These interventions enhance cardiopulmonary reserve, improve tissue oxygenation, reduce systemic inflammation, and promote better wound healing capacity. Importantly, prehabilitation may also improve long-term quality of life and postoperative independence, particularly among elderly or frail patients undergoing complex abdominal wall reconstruction.

Nutritional optimization represents a critical component of prehabilitation. Malnutrition is frequently underrecognized in ventral hernia patients, particularly in individuals with obesity, where sarcopenic obesity may coexist despite an elevated body mass index. Hypoalbuminemia and protein deficiency are strongly associated with increased postoperative morbidity and impaired fascial healing [19]. Current recommendations advocate assessment of nutritional status using serum markers, body composition analysis, and validated nutritional screening tools. High-protein supplementation, micronutrient correction, and dietitian-guided interventions have been shown to improve perioperative outcomes in high-risk surgical populations.

Smoking cessation is another essential strategy in preoperative optimization. Tobacco exposure impairs tissue perfusion, collagen synthesis, and immune function while increasing the risk of wound infection and hernia recurrence. Studies suggest that cessation of smoking at least four weeks before surgery significantly reduces postoperative complications [20]. Similarly, strict glycemic control in diabetic patients reduces surgical site infections and enhances wound healing by minimizing microvascular dysfunction and impaired leukocyte activity.

Exercise-based prehabilitation has also gained substantial attention. In a randomized controlled trial, Liang et al. demonstrated that structured prehabilitation programs significantly reduced surgical site infections and improved postoperative recovery compared with standard perioperative care [21]. Likewise, Renshaw et al. reported that supervised preoperative exercise regimens improve physical conditioning, reduce pulmonary complications, and facilitate earlier functional recovery following ventral hernia repair [22]. These exercise programs typically incorporate aerobic conditioning, resistance training, respiratory physiotherapy, and abdominal wall strengthening exercises tailored to individual patient needs.

Psychological optimization is another emerging dimension of prehabilitation. Chronic ventral hernias often lead to impaired body image, anxiety, depression, and reduced quality of life. Preoperative counseling and expectation management improve patient satisfaction and adherence to postoperative rehabilitation protocols. Enhanced patient education regarding operative risks, recovery timelines, lifestyle modification, and recurrence prevention further contributes to improved long-term outcomes.

The role of multidisciplinary care pathways has become increasingly important in complex abdominal wall reconstruction. Specialized multidisciplinary teams (MDTs) commonly include general surgeons, plastic surgeons, anesthesiologists, radiologists, physiotherapists, nutritionists, pain specialists, and wound care experts. Such collaborative models facilitate comprehensive assessment, individualized risk stratification, and coordinated perioperative management [23]. Studies evaluating MDT-based hernia programs have demonstrated reductions in postoperative complications, recurrence rates, and hospital length of stay when compared with conventional care models.

Enhanced recovery after surgery (ERAS) protocols have also been integrated into ventral hernia management. ERAS pathways emphasize multimodal analgesia, early mobilization, opioid-sparing strategies, optimized fluid management, early enteral nutrition, and standardized perioperative care. These protocols reduce physiological stress responses and improve recovery while minimizing postoperative morbidity [24]. The combination of ERAS principles with prehabilitation represents a comprehensive perioperative optimization strategy in modern abdominal wall reconstruction.

Importantly, prehabilitation also contributes to surgical decision-making. In selected patients with morbid obesity or severe metabolic dysfunction, delayed elective repair following weight reduction or bariatric intervention may provide superior outcomes compared with immediate surgery. Similarly, aggressive optimization may convert initially high-risk patients into acceptable operative candidates, thereby expanding treatment opportunities while reducing perioperative risk.

Overall, prehabilitation and multidisciplinary optimization have transformed ventral hernia surgery from a purely procedural intervention into a comprehensive perioperative care model focused on functional restoration, risk reduction, and long-term patient-centered outcomes.

Adjunct techniques in complex hernia repair

Management of giant ventral hernias and defects associated with significant loss of domain (LOD) remains one of the most challenging aspects of abdominal wall reconstruction. In such patients, chronic herniation leads to progressive retraction and lateralization of the abdominal wall musculature, reduction in intra-abdominal cavity volume, and adaptive changes in respiratory mechanics [25]. Attempting primary fascial closure under tension in these cases may result in respiratory compromise, abdominal compartment syndrome, impaired tissue perfusion, and increased recurrence risk. Consequently, several adjunctive techniques have been developed to facilitate tension-free closure and improve surgical outcomes in complex hernia repair.

Botulinum toxin A (BTA) injection has emerged as an important preoperative adjunct in abdominal wall reconstruction. BTA acts by producing temporary flaccid paralysis of the lateral abdominal wall muscles, including the external oblique, internal oblique, and transversus abdominis muscles. This chemical denervation results in elongation and medialization of the musculofascial components, thereby reducing fascial tension during closure [26].

Typically administered under ultrasound guidance approximately 4-6 weeks before surgery, BTA has demonstrated favorable effects on fascial closure rates and abdominal wall compliance. A meta-analysis by Timmer et al. reported that preoperative BTA significantly improves primary fascial closure rates while reducing the need for extensive component separation procedures [21]. Systematic reviews further suggest that BTA decreases operative tension, facilitates minimally invasive reconstruction, and may reduce postoperative pain [22]. The technique is particularly valuable in giant ventral hernias, recurrent defects, and patients with severe muscular retraction.

Progressive pneumoperitoneum (PPP) represents another important adjunctive strategy in patients with severe LOD. Initially described decades ago, PPP involves gradual insufflation of air into the peritoneal cavity over several days to weeks prior to definitive repair [27]. This progressive expansion increases intra-abdominal capacity, stretches the abdominal wall musculature, and allows physiological adaptation of the diaphragm and respiratory system to increased intra-abdominal volume.

PPP has been associated with improved tolerance of fascial closure and reduced postoperative respiratory compromise in complex hernia repair. Additionally, it facilitates the reduction of chronically herniated viscera into the abdominal cavity while minimizing tension on reconstructed tissues. Although complications such as subcutaneous emphysema, pneumothorax, and catheter-related infections may occur, contemporary studies demonstrate acceptable safety profiles when performed in experienced centers [27].

More recently, the combined use of BTA and PPP has gained increasing popularity. This combined strategy simultaneously increases abdominal wall compliance and expands the intra-abdominal domain, thereby optimizing conditions for tension-free closure. A systematic review by Giuffrida et al. demonstrated favorable outcomes with combined BTA and PPP in patients with giant ventral hernias, including improved fascial closure rates and reduced postoperative complications [24]. These approaches have significantly expanded reconstructive possibilities in previously inoperable or extremely high-risk hernias.

Additional adjunctive techniques include tissue expanders, temporary abdominal wall traction systems, and vacuum-assisted closure devices. Although less commonly utilized, these strategies may provide benefit in selected cases requiring staged reconstruction or management of contaminated abdominal wall defects.

The increasing adoption of adjunctive preoperative techniques reflects the broader evolution of abdominal wall reconstruction toward individualized, physiology-based management strategies aimed at maximizing fascial closure while minimizing postoperative morbidity.

Open versus laparoscopic repair

The optimal surgical approach for ventral hernia repair remains an area of ongoing debate and investigation. Both open and laparoscopic techniques are widely practiced, with selection depending on defect characteristics, patient comorbidities, contamination status, surgeon expertise, and available institutional resources. Over the past two decades, minimally invasive approaches have gained increasing popularity because of their potential advantages in reducing wound morbidity and enhancing postoperative recovery [25].

Open ventral hernia repair remains an essential technique, particularly for large, recurrent, incarcerated, or complex defects requiring extensive abdominal wall reconstruction. Open approaches permit wide dissection, component separation, retromuscular mesh placement, and management of significant adhesions or contaminated fields. However, open surgery is associated with larger incisions, increased tissue trauma, greater postoperative pain, prolonged recovery, and higher rates of wound complications, including surgical site infection, seroma formation, and skin necrosis [26].

Laparoscopic ventral hernia repair was introduced to reduce these complications while maintaining acceptable recurrence outcomes. A landmark Cochrane review demonstrated that laparoscopic repair significantly reduces wound infection rates and shortens hospital stay compared with open surgery, while recurrence rates remain comparable between the two techniques [25]. Minimally invasive repair minimizes soft tissue dissection and preserves abdominal wall vascularity, thereby reducing wound-related morbidity.

Subsequent meta-analyses have consistently confirmed these findings. Martins et al. reported reduced postoperative pain, fewer surgical site occurrences, earlier ambulation, and faster return to normal activity with laparoscopic approaches [26]. These benefits are particularly important in obese patients, who are at increased risk of wound complications following open surgery. Additionally, laparoscopic techniques may reduce postoperative adhesions and facilitate earlier recovery.

Despite these advantages, laparoscopic repair also presents important limitations. Operative times are often longer, especially during the learning curve phase. Intraperitoneal mesh placement may predispose to adhesions, bowel erosion, fistula formation, and chronic pain related to fixation devices [27]. Furthermore, laparoscopic repair may be technically challenging in patients with large defects, severe adhesions, multiple prior surgeries, or significant loss of domain.

The intraperitoneal onlay mesh (IPOM) technique became the most widely adopted laparoscopic method for ventral hernia repair. However, concerns regarding intraperitoneal mesh-related complications stimulated the development of modified approaches. Registry-based studies comparing laparoscopic IPOM with open sublay repair have demonstrated similar recurrence rates but lower wound complication rates with minimally invasive repair [28].

The IPOM-plus technique, which incorporates primary fascial defect closure prior to mesh placement, has further improved outcomes. Meta-analyses demonstrate that fascial closure reduces seroma formation, mesh bulging, and recurrence while restoring abdominal wall function more effectively than bridged repairs [29]. Consequently, defect closure has become increasingly favored whenever technically feasible.

Hybrid intraperitoneal onlay mesh (Hybrid IPOM) repair combines the advantages of open and laparoscopic techniques by incorporating limited open adhesiolysis and fascial defect closure with laparoscopic mesh placement. This approach may be particularly useful in large or complex ventral hernias where complete laparoscopic repair is technically challenging. Recent studies have reported favorable outcomes with Hybrid IPOM, including reduced wound morbidity, improved fascial closure rates, and acceptable recurrence rates, making it a valuable option in selected patients [30].

Patient selection remains critical in determining the optimal surgical approach. Small-to-moderate uncomplicated hernias are often ideally suited for laparoscopic repair, whereas giant defects, extensive loss of domain, active contamination, or the need for advanced reconstruction may favor open or hybrid approaches. Contemporary practice increasingly emphasizes individualized treatment algorithms rather than a universal preference for any single technique.

Robotic ventral hernia repair

Robotic-assisted ventral hernia repair represents one of the most significant technological advancements in contemporary abdominal wall surgery. The robotic platform was developed to overcome several technical limitations of conventional laparoscopy, particularly restricted instrument articulation, ergonomic discomfort, and limited intracorporeal suturing capability [31].

Robotic systems provide high-definition three-dimensional visualization, tremor filtration, wristed instrumentation, and enhanced dexterity, facilitating precise dissection and complex reconstructive maneuvers. These advantages are particularly beneficial in retromuscular dissection, intracorporeal suturing, and minimally invasive component separation procedures.

Systematic reviews have demonstrated that robotic ventral hernia repair is associated with reduced postoperative pain, shorter hospitalization, lower wound morbidity, and faster recovery compared with open surgery [31]. Robotic approaches also enable minimally invasive retromuscular mesh placement and fascial closure, thereby combining the biomechanical advantages of open reconstruction with the reduced wound morbidity of minimally invasive surgery.

Robotic transabdominal preperitoneal (r-TAPP) repair has shown favorable outcomes in selected ventral hernias. Feasibility studies by Orthopoulos and Kudsi demonstrated safe mesh placement within the preperitoneal plane with low complication rates and satisfactory early outcomes [32]. Similarly, robotic retromuscular repairs and robotic TAR procedures have gained popularity for complex abdominal wall reconstruction.

Clinical series evaluating robotic ventral hernia repair report low recurrence rates, reduced wound complications, and high patient satisfaction [33]. Robotic TAR, in particular, has expanded the feasibility of minimally invasive reconstruction for large and recurrent hernias that previously required open surgery.

Nevertheless, several limitations remain. Robotic procedures generally involve longer operative times and substantially higher costs compared with conventional laparoscopy [31]. Access to robotic platforms may also be limited in resource-constrained healthcare settings. Furthermore, long-term recurrence data remain relatively limited because many robotic techniques are still evolving.

Despite these concerns, robotic surgery continues to gain momentum due to its technical versatility and expanding indications. Ongoing advances in robotic technology, instrument design, and surgeon experience are likely to further refine its role in abdominal wall reconstruction.

Advanced minimally invasive techniques

The evolution of minimally invasive abdominal wall surgery has led to the development of advanced reconstructive techniques designed to combine the advantages of retromuscular mesh placement with reduced surgical trauma. Among these innovations, enhanced-view totally extraperitoneal (eTEP) repair has emerged as a major advancement in ventral hernia surgery [34].

The eTEP approach enables access to the retromuscular plane using minimally invasive techniques while avoiding intraperitoneal mesh placement. This strategy combines the biomechanical advantages of the Rives-Stoppa repair with the reduced wound morbidity associated with laparoscopy. By placing mesh within the retromuscular space, eTEP minimizes bowel contact, reduces adhesion formation, and avoids complications associated with intraperitoneal prostheses.

Early studies have demonstrated the feasibility, safety, and favorable postoperative outcomes associated with eTEP repair [35]. Patients undergoing eTEP often experience reduced postoperative pain, shorter hospitalization, and improved cosmetic outcomes compared with open reconstruction. Furthermore, the technique facilitates wide mesh overlaps and tension-free fascial closure.

Transversus abdominis release (TAR) represents another transformative advancement in abdominal wall reconstruction. Initially described by Novitsky et al., TAR involves division of the transversus abdominis muscle to allow medial advancement of the abdominal wall musculature and creation of a large retromuscular space for mesh placement [36].

TAR has become particularly valuable in giant ventral hernias, recurrent defects, and complex abdominal wall reconstruction because it permits restoration of midline fascial continuity while minimizing wound morbidity. Studies report low recurrence rates, favorable functional outcomes, and acceptable complication profiles following TAR [36,37]. The technique may be performed using open, laparoscopic, or robotic approaches, with robotic TAR increasingly gaining popularity.

These advanced techniques reflect the broader trend toward functional abdominal wall reconstruction focused on restoring anatomy, biomechanics, and patient quality of life rather than simply achieving defect closure.

Component separation and reconstruction techniques

Component separation techniques constitute a cornerstone of modern complex abdominal wall reconstruction. These procedures are designed to mobilize musculofascial layers of the abdominal wall to facilitate tension-free midline closure in large defects that cannot otherwise be approximated primarily [38].

The original anterior component separation technique involves the release of the external oblique aponeurosis to permit the medial advancement of the rectus complex. Anatomical studies have demonstrated substantial increases in abdominal wall compliance and fascial mobility following component separation [38]. However, traditional open anterior component separation requires extensive subcutaneous dissection and is associated with high wound complication rates.

To overcome these limitations, endoscopic and minimally invasive component separation techniques were subsequently developed. Meta-analyses demonstrate that endoscopic component separation significantly reduces wound morbidity while maintaining effective fascial advancement [39]. Preservation of perforator vessels and minimization of skin flap dissection contribute to improved tissue perfusion and reduced infection rates.

Posterior component separation with TAR has increasingly supplanted traditional anterior approaches in many specialized centers because it allows extensive medialization without large skin flaps while providing a large retromuscular plane for mesh placement [36].

The Rives-Stoppa repair remains one of the most important reconstructive principles in abdominal wall surgery. This retromuscular mesh technique provides durable reinforcement with excellent biomechanical properties and low recurrence rates [40]. Meta-analyses consistently support retromuscular mesh placement as one of the most effective methods for ventral hernia repair.

Complex abdominal wall reconstruction increasingly emphasizes restoration of functional anatomy, abdominal wall dynamics, and quality of life rather than merely prevention of recurrence. Consequently, reconstructive strategies are becoming progressively individualized according to defect morphology, patient physiology, contamination status, and surgeon expertise.

Perioperative strategies and the role of drains

Optimal perioperative management plays a major role in determining outcomes following ventral hernia repair. Strategies aimed at minimizing surgical site occurrences, seroma formation, postoperative pain, and respiratory complications continue to evolve as evidence accumulates.

The use of surgical drains remains one of the most controversial aspects of perioperative care in abdominal wall reconstruction. Traditionally, drains were routinely placed to prevent fluid accumulation, seroma formation, and hematoma development following extensive dissection or mesh placement. However, concerns have emerged regarding increased infection risk, patient discomfort, prolonged hospitalization, and delayed recovery associated with drain use [41].

Randomized controlled trials evaluating drain placement have produced conflicting findings. Willemin et al. demonstrated no significant difference in postoperative complications between the drain and no-drain groups following ventral hernia repair [41]. Similarly, a large registry-based analysis involving more than 39,000 patients found no clear overall benefit associated with routine drain usage [42].

Current evidence, therefore, suggests that routine prophylactic drain placement may not be universally necessary. Instead, drain use should be individualized according to factors such as extent of dissection, size of dead space, contamination risk, mesh position, and surgeon preference. Drains may still provide benefit in selected complex reconstructions involving extensive soft tissue mobilization or large retromuscular spaces.

Other perioperative strategies, including multimodal analgesia, early mobilization, thromboembolism prophylaxis, optimized fluid therapy, and enhanced recovery pathways, contribute significantly to improved postoperative outcomes. Contemporary abdominal wall reconstruction, therefore, increasingly relies on standardized perioperative protocols integrated within multidisciplinary care systems.

Emerging concepts, future directions, and personalized approaches in ventral hernia repair

The field of ventral hernia surgery is undergoing rapid transformation with increasing emphasis on precision-based abdominal wall reconstruction, functional restoration, and long-term patient-centered outcomes. Although significant progress has been achieved in mesh technology, minimally invasive surgery, and perioperative optimization, recurrence, chronic postoperative pain, mesh-related complications, and healthcare expenditure remain major challenges worldwide. Contemporary research, therefore, increasingly focuses on individualized treatment strategies, advanced reconstructive techniques, biologic understanding of abdominal wall failure, and technology-driven innovations aimed at improving both functional and anatomical outcomes.

One of the most important emerging concepts in modern abdominal wall surgery is the transition from simple “hernia repair” toward comprehensive “abdominal wall reconstruction.” Traditional surgical management primarily emphasized fascial closure and recurrence prevention; however, current reconstructive philosophy prioritizes restoration of abdominal wall physiology, musculofascial continuity, respiratory dynamics, and biomechanical integrity [43]. This shift reflects increasing recognition that successful ventral hernia surgery should not only prevent recurrence but also restore functional abdominal wall support, improve mobility, and enhance overall quality of life.

Recent evidence highlights the growing importance of patient-reported outcomes in evaluating surgical success. Lodha et al. demonstrated significant improvement in quality-of-life parameters following ventral hernia repair, including reductions in pain scores, enhanced physical functioning, and improved body image perception [44]. Their study reported substantial postoperative improvement in patient-reported functional status and daily activity performance, emphasizing that modern outcome assessment should extend beyond recurrence rates alone. Chronic pain and impaired functional capacity remain major determinants of patient dissatisfaction despite technically successful repairs, reinforcing the importance of functional reconstruction.

Increasing emphasis has also been placed on standardized classification systems and individualized surgical planning. The European Hernia Society classification proposed by Muysoms et al. has significantly improved consistency in reporting ventral hernia characteristics by incorporating parameters such as defect size, anatomical location, and recurrence status [45]. Standardized classification facilitates better comparison between studies, improves operative planning, and enables more tailored treatment algorithms according to patient-specific and defect-specific factors.

Advanced preoperative imaging has become integral to complex abdominal wall reconstruction. Computed tomography-based volumetric analysis enables accurate assessment of defect dimensions, abdominal wall musculature, and loss of domain (LOD), thereby facilitating risk stratification and operative planning. Bueno-Lledó et al. demonstrated that combined preoperative botulinum toxin A (BTA) administration and progressive pneumoperitoneum (PPP) significantly improve closure feasibility in giant ventral hernias with LOD [46]. Their study reported high fascial closure rates and reduced postoperative respiratory complications, highlighting the importance of preoperative anatomical optimization in complex reconstruction.

Biomechanical principles increasingly influence mesh selection and placement strategies. Retromuscular mesh placement is currently regarded as one of the most effective reconstructive approaches because it provides favorable force distribution, improved mesh incorporation, and reduced recurrence risk. Theis et al. emphasized the importance of retromuscular mesh positioning in optimizing long-term outcomes and minimizing mesh-related complications [47]. Similarly, a network meta-analysis by Holihan et al. demonstrated that sublay and retromuscular repairs are associated with lower recurrence and surgical site complication rates compared with onlay mesh placement [48]. These findings have reinforced the widespread adoption of retromuscular reconstruction in both open and minimally invasive surgery.

Prophylactic mesh reinforcement represents another evolving strategy in abdominal wall surgery. Incisional hernia remains a major complication following midline laparotomy, particularly among high-risk patients with obesity, smoking history, abdominal aortic aneurysm, or multiple prior surgeries. A meta-analysis by Henriksen et al. demonstrated that prophylactic mesh augmentation significantly reduces the incidence of postoperative incisional hernia formation without major increases in wound complications [49]. Consequently, prophylactic mesh placement is increasingly recommended in selected high-risk patient populations to prevent future abdominal wall failure.

Technological innovation continues to expand the role of minimally invasive surgery in ventral hernia repair. Robotic-assisted reconstruction has gained significant popularity because of enhanced dexterity, improved intracorporeal suturing capability, and superior three-dimensional visualization. Standardization efforts proposed by Halpern et al. aim to improve reproducibility and consistency in robotic abdominal wall reconstruction procedures [50]. Robotic surgery facilitates advanced reconstructive techniques such as minimally invasive transversus abdominis release (TAR), retrorectus repair, and posterior component separation while minimizing wound morbidity.

TAR has emerged as one of the most important advancements in complex abdominal wall reconstruction. Novitsky et al. initially described TAR as a posterior component separation technique enabling wide retromuscular mesh placement and tension-free midline closure [51]. Subsequent long-term outcome studies by Winder et al. demonstrated durable repair outcomes with low recurrence rates and acceptable morbidity profiles following TAR procedures [52]. These studies reported recurrence rates generally below 10% during extended follow-up, supporting TAR as a highly effective technique for large and recurrent ventral hernias.

The integration of multidisciplinary abdominal wall reconstruction programs has further transformed ventral hernia management. Specialized centers increasingly incorporate surgeons, radiologists, anesthesiologists, physiotherapists, nutritionists, wound care specialists, and pain management teams into coordinated treatment pathways. Such multidisciplinary models facilitate comprehensive prehabilitation, individualized operative planning, enhanced recovery protocols, and long-term functional rehabilitation. Evidence suggests that these structured pathways reduce postoperative complications, improve patient satisfaction, and optimize resource utilization.

Emerging technologies such as artificial intelligence (AI), machine learning, and three-dimensional surgical planning may further redefine abdominal wall reconstruction in the future. AI-based predictive analytics may help identify patients at increased risk of recurrence, surgical site infection, or chronic pain. Similarly, three-dimensional printing and virtual surgical simulation may improve planning in giant or recurrent hernias requiring complex reconstruction. Smart biomaterials and bioengineered prostheses capable of controlled tissue integration and anti-inflammatory modulation are also under investigation as next-generation mesh technologies.

Despite major advances, several controversies remain unresolved in ventral hernia surgery. Optimal mesh selection in contaminated fields, long-term outcomes of biosynthetic materials, indications for robotic surgery, management of chronic postoperative pain, and cost-effectiveness of advanced technologies continue to be debated. Many contemporary studies are also limited by heterogeneity in patient populations, inconsistent outcome reporting, and relatively short follow-up periods.

Future research should therefore focus on multicenter randomized controlled trials evaluating long-term recurrence, abdominal wall function, quality-of-life outcomes, and the economic impact of emerging reconstructive strategies. Standardization of reporting systems and incorporation of validated patient-reported outcome measures will be essential for improving evidence quality and facilitating meaningful comparison between operative techniques.

Overall, ventral hernia repair has evolved into a highly specialized field integrating advanced reconstructive principles, minimally invasive technology, multidisciplinary perioperative optimization, and individualized patient-centered care. Continued innovation in biomaterials, surgical techniques, imaging, and perioperative management is likely to further improve outcomes and redefine the future landscape of abdominal wall reconstruction.

Controversies and challenges in contemporary ventral hernia repair

Despite remarkable advances in abdominal wall reconstruction, several controversies and unresolved challenges continue to influence decision-making in ventral hernia surgery. Persistent concerns regarding recurrence, mesh-related complications, chronic pain, infection risk, operative costs, and optimal reconstructive strategy highlight the complexity of ventral hernia management and the ongoing need for evidence-based refinement of surgical techniques.

One of the most debated issues remains the optimal choice of prosthetic material, particularly in contaminated or potentially contaminated surgical fields. Historically, biologic meshes were favored in contaminated environments because of concerns regarding synthetic mesh infection and explantation. However, increasing evidence challenges this traditional paradigm. Atema et al., in a systematic review evaluating complex abdominal wall reconstruction in contaminated fields, demonstrated that synthetic mesh can be safely utilized with acceptable infection rates and significantly lower recurrence compared with biologic materials [53]. Similarly, findings from the RICH study by Itani et al. evaluated biologic porcine tissue matrices in contaminated ventral hernia repair and demonstrated acceptable short-term outcomes, although recurrence and cost concerns remained substantial [54]. These studies have fueled ongoing debate regarding the routine use of expensive biologic prostheses when synthetic alternatives may offer superior long-term durability at lower cost.

Mesh position also remains a critical determinant of postoperative outcomes and an important area of controversy. Numerous studies have compared onlay, inlay, sublay, intraperitoneal, and retromuscular mesh placements with varying conclusions. Contemporary evidence increasingly favors retromuscular and sublay positions because of their superior biomechanical characteristics and lower recurrence rates. Bittner et al. and Sosin et al. highlighted that retromuscular mesh placement provides improved tissue integration and reduced postoperative complications compared with alternative planes [55,56]. Likewise, Holihan et al. demonstrated lower recurrence rates with sublay techniques when compared with onlay repair [48]. These findings have contributed to the growing preference for retromuscular reconstruction in modern abdominal wall surgery.

Another ongoing challenge involves the management of complex ventral hernias with significant loss of domain and extensive musculofascial retraction. Posterior component separation and transversus abdominis release (TAR) have substantially improved reconstructive capability in such patients. Carbonell et al. first emphasized the utility of posterior component separation in facilitating large retromuscular repairs while minimizing extensive skin flap dissection [57]. Nevertheless, these procedures remain technically demanding and are associated with prolonged operative times, increased resource utilization, and potential neurovascular injury. Consequently, careful patient selection and specialized surgical expertise remain essential.

Minimally invasive ventral hernia repair has evolved considerably over the last decade, yet debate persists regarding the relative benefits of laparoscopic versus robotic approaches. Laparoscopic repair offers reduced wound morbidity, shorter hospitalization, and faster postoperative recovery, but limitations in intracorporeal suturing and ergonomics may restrict its application in complex reconstructions. Robotic-assisted surgery was introduced to overcome these technical constraints. Warren et al. compared laparoscopic and robotic retromuscular ventral hernia repair and demonstrated improved technical feasibility and reconstructive flexibility with robotic surgery [58]. Furthermore, Carbonell et al. reported reduced hospital length of stay following robotic-assisted retromuscular reconstruction compared with conventional approaches [59].

Despite these advantages, robotic surgery remains controversial because of increased operative costs and prolonged operating times. Acquisition and maintenance expenses associated with robotic systems may limit accessibility, particularly in low-resource healthcare settings. Additionally, long-term recurrence data for many robotic techniques remain limited because these procedures are relatively recent innovations. Questions, therefore, remain regarding cost-effectiveness and broader applicability of robotic reconstruction.

Primary fascial closure during minimally invasive ventral hernia repair has also become an important topic of investigation. Earlier laparoscopic techniques often utilized bridged repairs without defect closure, which were associated with seroma formation, mesh bulging, and recurrence. Bernardi et al., in a systematic review and meta-analysis, demonstrated that primary fascial closure during laparoscopic ventral hernia repair significantly improves postoperative outcomes and reduces seroma formation [60]. Consequently, IPOM-plus and other defect closure strategies have gained widespread acceptance in contemporary minimally invasive reconstruction.

Prevention of incisional hernia formation remains another major focus in abdominal wall surgery. Technical aspects of abdominal wall closure play a pivotal role in long-term fascial integrity. The STITCH trial conducted by Deerenberg et al. demonstrated that small-bite fascial closure significantly reduces the incidence of incisional hernia compared with traditional large-bite techniques [61]. These findings have substantially influenced modern abdominal wall closure guidelines and reinforced the importance of meticulous surgical techniques in hernia prevention.

The role of surgical drains following ventral hernia repair also remains controversial. Drains have traditionally been used to prevent seroma formation and fluid accumulation following extensive dissection; however, concerns regarding infection risk and delayed recovery persist. Krpata et al., using data from the Americas Hernia Society Quality Collaborative, demonstrated that drain placement after retromuscular ventral hernia repair with synthetic mesh does not significantly increase infectious complications [62]. Nevertheless, evidence remains inconsistent, and current practice increasingly favors individualized drain usage rather than routine placement.

Another major challenge in ventral hernia surgery involves chronic postoperative pain and impaired quality of life. Although recurrence prevention remains important, chronic discomfort, stiffness, foreign-body sensation, and reduced abdominal wall compliance may significantly impair long-term patient satisfaction. Mesh weight, fixation techniques, operative plane, and nerve injury all contribute to postoperative pain syndromes. Lightweight meshes and atraumatic fixation methods have therefore gained increasing attention as strategies to minimize chronic pain while preserving repair durability.

Obesity continues to represent one of the most important predictors of adverse outcomes following ventral hernia repair. Increased intra-abdominal pressure, impaired tissue healing, diabetes, and metabolic dysfunction collectively increase recurrence risk and wound complications in obese patients. Consequently, prehabilitation and weight reduction strategies are increasingly emphasized before elective abdominal wall reconstruction. However, delaying repair for prolonged optimization may increase the risk of incarceration and symptom progression, creating additional management dilemmas.

Healthcare economics also represent an increasingly important issue in ventral hernia management. Advanced technologies, including robotic surgery, biologic mesh, biosynthetic materials, and prolonged prehabilitation programs, substantially increase procedural costs. Although these approaches may improve short-term outcomes in selected patients, robust evidence regarding long-term cost-effectiveness remains limited. Future healthcare policy decisions will therefore require a careful balance between technological innovation, patient benefit, and resource allocation.

Another unresolved area involves the lack of standardized outcome reporting across hernia studies. Variability in recurrence definitions, follow-up duration, patient-reported outcomes, and complication reporting limits direct comparison between surgical techniques and prosthetic materials. Increasing adoption of standardized registries and collaborative databases such as the Americas Hernia Society Quality Collaborative may help improve evidence quality and facilitate more meaningful comparative analyses in future research.

Overall, contemporary ventral hernia surgery continues to evolve within a landscape of ongoing innovation, controversy, and technological advancement. While substantial progress has improved reconstructive outcomes and expanded surgical possibilities, persistent challenges related to recurrence, chronic pain, mesh selection, contamination, and healthcare costs underscore the need for continued research and individualized patient-centered management strategies.

Key takeaways

Mesh-based reinforcement remains the cornerstone of ventral hernia repair, with synthetic meshes demonstrating favorable long-term outcomes in most clinical settings. Preoperative optimization, including weight management, smoking cessation, nutritional support, and prehabilitation, plays a critical role in reducing postoperative complications. Minimally invasive approaches, including laparoscopic and robotic techniques, are associated with reduced wound morbidity and faster recovery in appropriately selected patients. Advanced reconstructive strategies such as transversus abdominis release (TAR), enhanced-view totally extraperitoneal (eTEP) repair, and adjunctive techniques for loss of domain have expanded treatment options for complex hernias. Contemporary ventral hernia management increasingly emphasizes individualized, multidisciplinary, and patient-centered care aimed at optimizing both functional and long-term outcomes.

## Conclusions

Ventral hernia repair has evolved into a complex and dynamic field, integrating advances in surgical techniques, biomaterials, and perioperative care. High-quality evidence supports the use of synthetic mesh over biologic mesh in most scenarios, with lightweight materials offering improved patient comfort. Minimally invasive approaches, including laparoscopic and robotic techniques, provide significant benefits in reducing complications and enhancing recovery.

Adjunct techniques such as botulinum toxin and progressive pneumoperitoneum have improved outcomes in complex cases, while advanced approaches such as eTEP and TAR continue to refine surgical practice. Prehabilitation and multidisciplinary care play a crucial role in optimizing outcomes. Future research should focus on long-term outcomes, cost-effectiveness, and personalized treatment strategies to further improve patient care in ventral hernia repair.
